# Cybersecurity of Hospitals: discussing the challenges and working towards mitigating the risks

**DOI:** 10.1186/s12911-020-01161-7

**Published:** 2020-07-03

**Authors:** Salem T. Argaw, Juan R. Troncoso-Pastoriza, Darren Lacey, Marie-Valentine Florin, Franck Calcavecchia, Denise Anderson, Wayne Burleson, Jan-Michael Vogel, Chana O’Leary, Bruce Eshaya-Chauvin, Antoine Flahault

**Affiliations:** 1 Institute of Global Health, Faculty of Medicine, University of Geneva, Campus Biotech, Chemin des Mines 9, 1202 Geneva, Switzerland; 2 School of Computer and Communication Sciences, EPFL (Ecole polytechnique fédérale de Lausanne), EPFL IC IINFCOM LDS, BC 266 (Bâtiment BC), Station 14, CH-1015 Lausanne, Switzerland; 3 Johns Hopkins University/Johns Hopkins Medicine, 5801 Smith Avenue, Davis Building, Suite 3110B, Baltimore, MD 21209 USA; 4 International Risk Governance Center (IRGC), EPFL (Ecole polytechnique fédérale de Lausanne), EPFL ENT-R IRGC, BAC 001.1 (Château de Bassenges), Station 5, CH-1015 Lausanne, Switzerland; 5 Hôpitaux Universitaires de Genève, Rue Gabrielle-Perret-Gentil 4, CH-1211 Genève 14, Switzerland; 6 National Health Information Sharing and Analysis Center (NH-ISAC), 226 North Nova Road, Suite 391, Ormond Beach, Florida, 32174 USA; 7 Electrical and Computer Engineering, University of Massachusetts Amherst, 309B Knowles Engineering Bldg, University of Massachusetts, 151 Holdsworth Way, Amherst, MA 01003-9284 USA; 8 Department of Information Technology, Charité-Universitätsmedizin Berlin, Charitéplatz 1, 10117 Berlin, Germany; 9 Aspen University, 1660 S. Albion St., Suite 525, Denver, Colorado, 80222 USA

## Executive summary

The increasing incorporation of technology into the health field is leading to greater precision in healthcare; however, advancements in cybersecurity measures are still required. According to a 2016 report by IBM and the Ponemon Institute, the frequency of data breaches in the healthcare industry has been rising since 2010 [1], and it is now among the sectors most targeted by cyberattacks globally [2]. Due to its immutability, the information accessed through health data breaches is of particular interest to criminals [3]. Blood type, past surgeries and diagnoses, and other personal health information are contained in an individual’s medical file. As these records include private data such as name, date of birth, insurance and health provider information, as well as health and genetic information, it is not possible to restore privacy or to reverse psychosocial harm when private data are compromised.

These sorts of attacks are not only a threat to patients’ identity and finances, but they can also impede hospital operations and place the health and well-being of patients at risk. The United Kingdom’s National Health System hospitals, which suffered from the WannaCry ransomware attacks in May 2017, were forced to delay treatment plans and even to reroute incoming ambulances because they lost access to hospital information systems [4]. Among these operational delays and the financial consequences of data breaches and ransomware attacks, cyberattacks have long-term detrimental effects on the reputation and revenue of hospitals and health facilities.

In response to these global attacks, the *M8 Alliance* undertook a project that began with a scoping review on cyberattacks against hospitals [5]. The review was a basis for several teleconferences conducted by a multidisciplinary team of experts. A workshop ensued in April 2018 at the bi-annual *Geneva Health Forum* (GHF). The purpose of these meetings was to exchange perceived threats, to promote interdisciplinary discussion, and to propose practical recommendations for hospitals across the globe. The onsite meeting at the GHF was organized as a *World Health Summit Expert Meeting* on the cybersecurity of hospitals [6].

Here, we describe the most prominent discussions and recommendations from this working group for other security officers, hospital decision makers, vendors, manufacturers, industry representatives, and academics in the field. We begin with some case examples that serve to illustrate what these attacks look like and how health organizations have responded in the past. We then discuss the need to address cybersecurity through the product lifecycle in a preventative and proactive way as well as an approach to cybersecurity that values quality IT at the foundation with a stable application base and strong IT infrastructure. A risk-based approach is recommended, beginning with the identification of at-risk IT assets, followed by management of tradeoffs between risks and benefits, as well as different types of risks. The training of end-users is emphasized, alongside strategies such as vulnerability management and patch management, the controlled and restrictive granting of administrative privileges, and the development of incident response and business continuity plans. Information sharing between stakeholders is also recommended in order to build resilience. We conclude with a discussion on privacy-conscious data sharing and the unique challenges medical devices pose to security.

## Introduction

*Personally identifiable information* (PII) and *protected health information* (PHI) are handled by almost every department in a hospital, in one or more health information system. All healthcare providers (e.g., physicians, physician assistants, nurses, pharmacists, technicians, dietitians, physical therapists) use electronic health records (EHR), e-Prescribing software, remote patient monitoring, and/or laboratory information systems; the billing office works with insurance and financial information through medical billing software; scheduling and administration departments work with clinical data on scheduling software, and the list continues. While PII in organizations within most other fields (e.g., academic institutions or businesses) are typically contained within limited departments where cybersecurity measures can be centralized, in a hospital setting, the data are highly sensitive and valuable, yet almost all departments handle it at least in some manner. Cybersecurity measures aim to protect PII and PHI by securing devices, electronic systems, networks, and data from attacks.

In other fields, such as the financial sector the issue of cybersecurity has been confronted for decades, hence they have established policies and dedicated resources to invest in security, whereas the health field struggles to give sufficient attention and resources to the problem, as it is relatively new to this field. As healthcare is extremely cost constrained, very limited resources are allocated to IT security. Despite these constraints, cybersecurity in hospitals must take into account the thousands of interconnected medical devices and the often-inconsistent business processes. Connected medical devices introduce numerous vulnerabilities in a hospital’s cybersecurity; nevertheless, these devices are used throughout the hospital and can even be used off-site. The business process in hospitals can vary significantly from patient to patient, and is difficult to computationally model, this often requires openness (for data interoperability and access to health records in case of emergency), and hence, insecure codes.

Cybersecurity in the health field is unique due to the type of information at risk and the consequences for patient safety. When a credit card number is stolen, the bank cancels the card, issues a new one, and reimburses the client. However, when a patient’s PHI is stolen, the patient cannot change, for example, their birthdate, blood type, and health and genetic information. Once stolen, health information is widely applicable and valuable for a range of crimes, from identity theft to medical fraud. An individual’s health information is valued significantly more on the dark web than their social security number or credit card number; it can sell for 10 to 20 times more than this type of data [7, 8].

The regulatory framework around PHI has been evolving over the past two decades. In the United States (US), the Health Insurance Portability & Accountability Act (HIPAA) was passed in 1996; it enforced the protection of health information usage, disclosure, storage, and transmission [9]. This was followed by the Health Information Technology for Economic and Clinical Health (HITECH) Act in 2009, which increased penalties for HIPAA violations, strengthened breach notification, and encouraged the meaningful use of electronic health records [10]. In 2016, the General Data Protection Regulation (GDPR) was adopted by the European Union (EU) to replace existing regulations, and it entered into force in May 2018. GDPR implements provisions and requirements pertaining to the PII of all EU citizens, including provisions for breach notification and penalty implementation [11]. Although the increasingly strict regulations pose technological and organizational challenges for health institutions, they are for the protection of data and the cybersecurity of hospitals, as well as the sake of patient safety.

Cyberattacks risk delay and disruption of sensitive hospital operations and place patients’ lives at risk. When the British National Health Service hospitals were attacked in the global WannaCry attack of May 2017 or in the Hollywood Presbyterian Medical Center attack of February 2016, surgeries had to be delayed and patients diverted to nearby hospitals [4]. Cyberattacks can threaten a wide variety of services within a hospital, from surgeries to drug delivery, by targeting advanced equipment such as blood-product refrigerators, imaging equipment, automated drug dispensers and electronic health records, as well as by targeting supporting critical systems such as heating, ventilation, and air conditioning (HVAC). When EHR integrity is compromised, or they are suddenly encrypted in an attack, such as ransomware, providers lose access to critical information (e.g., patient allergies, current medications, and comorbidities). Hospitals are especially at risk in extreme or conflict situations, where stealth malware can stay hidden in the system until conveniently activated, thus leading to severe consequences when healthcare is most urgent (e.g., following a natural or human-instigated disaster). Cyberattacks can also compromise the trust in a doctor-patient relationship, e.g., if data are breached [12].

Moreover, when PHI is stolen, or patients’ lives are put at risk in a cyberattack, it is often nearly impossible to pinpoint the guilty party. Digital forensics is a challenging task in a hospital setting. Data are already used by many services and, when medical devices are involved, few services are equipped to collect necessary traces, run intrusion detection, or forensic analyses. It is difficult to track down the attacker(s), even when a ransom is paid, especially when anonymous cryptocurrencies such as Bitcoin, Dash, Verge, Monero, or ZCash are used. The question of liability is also complex, as there are uncertainties in liability attribution (e.g. in software liability), hence problematic for those who run operations. Assigning responsibility can lead to an oppositional relationship between hospitals and manufacturers. Instead of working together to ensure the highest security practices, they can become competitors by trying to avoid responsibility. However, without assigning responsibility and liability, it is difficult to maintain accountability and effectively deter future attacks.

In 2016, IBM X-Force reported that the healthcare industry faced more cyberattacks than other industries, even surpassing the financial sector [13]. That same year, the Ponemon Institute announced that the frequency of data breaches and their annual economic impact had been rising since 2010 [1]. A 2017 report also averaged the global cost per stolen record to be the highest in the healthcare sector [14]. The case examples in the following section (II) provide concrete details of recent attacks on healthcare organizations.

## Case examples

The following cases of cybersecurity breaches exemplify the variety of attacks the healthcare field has faced in different parts of the world, consequences of these attacks, and steps organizations took in response.

### Lukaskrankenhaus Neuss (Germany)

Lukaskrankenhaus Neuss is a public hospital founded in 1911 in Neuss, Germany with 537 beds and 1400 employees. In February 2016, employees encountered various error messages from a ransomware attack initiated through a social-engineering tactic. In response, the hospital took servers and computer systems offline to assess and cleanse infected systems. In the meantime, staff resorted to using pen, paper, and fax machines to continue their work but needed to postpone high-risk procedures [15].

While the hospital did not receive a direct demand for money, they were given an email address to contact for further instructions. No attempt was made to contact the attackers as recommended by local authorities [15]. The hospital reported that its backup system was kept up-to-date and only a few hours of data were lost, but a backlog of handwritten records from when the computer systems were offline need to be integrated with the remainder of the EHR eventually [15]. The hospital’s spokesperson predicted it would take a few months before their workflow was back to the status quo [16]. There was no evidence that patient data were breached.

### South-eastern Norway regional health authority (Norway)

The South-Eastern Norway Regional Health Authority (South-East RHF) is a state-run region-specific organization of specialist hospitals and healthcare services created in 2002 alongside three other regional authorities. In January 2018, South-East RHF announced that the PHI and records of nearly 2.9 million people (more than half of the population of Norway) had been compromised [17]. It is suspected that a sophisticated criminal group from a foreign spy or state agency led the attack targeting both patient health data and the health service’s interaction with Norway’s armed forces [18]. The vulnerability is thought to have come from the legacy system, Windows XP [18]. While the organization had begun security measures to reduce the risks brought on by Windows XP along with a plan to phase it out, the attack took place before they could implement the security measures [19].

While this attack did not seem to pose risks to patient safety or delays in hospital operations, the event raised concerns about future attacks on health data for the purpose of political gain and served as a wake-up call for GDPR. Under GDPR, the organization would have had to notify those affected within 72 h, which it did not do [20].

### Hancock regional hospital (United States)

The Hancock Regional Hospital is a small (71 beds) non-profit hospital in Greenfield, Indiana founded in 1951. On January 11, 2018, Hancock Regional faced a ransomware attack by the malware SamSam [21]. The attack targeted a server in their emergency IT backup-system and spread through the electronic connection between the backup site, located miles from the main campus, and the server farm at the hospital [22]. It was later discovered that the hackers had permanently corrupted components of the backup files from many systems, except the electronic medical record backup files. Investigators found that the attack was conducted using Microsoft’s Remote Desktop Protocol as an entry point into the server and that the hackers had compromised a hardware vendor’s administrative account to initiate the attack [23].

Following the attack, the hospital’s IT team shut down all network and desktop systems. Nevertheless, hospital operations continued within the confines of their downtime procedures. Patients were not diverted, and the hospital did not shut down. The hackers demanded four Bitcoins (55,000 USD) for the ransom, and the hospital paid. IT staff then spent the next three-and-a-half days decrypting files and trying to get the system to run normally [22]. They found no evidence that patient data had been compromised. The CEO, Steve Long, stated that the attack was found to be a premeditated targeted attack on the healthcare facility, by a sophisticated criminal group, and published an article explaining their decision to pay the ransom [22].

## Recommended approach to Cybersecurity in healthcare

### Quality IT at the foundation

For a health facility to have a strong information security posture, it requires quality IT: at least a stable application base and IT infrastructure. This is especially difficult to achieve in healthcare settings due to a lack in human resources, restraints in the budget, a history of underinvestment, and the complex application space; nevertheless, it is crucial.

Although there are no established models or tools for a health facility to use in evaluating the quality of its IT, there are a few markers that can shed some light. For example, a health facility with a stable application base does not have helpdesk call-logs that are overwhelmed with break/fix requests and its IT staff is not preoccupied primarily with repairing malfunctioning or broken applications.

Equally important to IT quality is the state of the IT infrastructure. The infrastructure can include any related resources and services used to deliver and support IT services (e.g., hardware platforms, software applications, operating systems, and networking and telecommunication tools) [24]. Information security requires that the IT infrastructure has configuration management, change management, and logging and monitoring in place. At its core, configuration management aims to maintain an updated inventory of IT assets and the relationship between different components. According to the Information Technology Infrastructure Library (ITIL), this involves identifying and reporting each assets’ version and its associated components [25]. Although it is a daunting task, well-maintained configuration management boosts vulnerability management and patch management. The SANS Institute states that “configuration management underlies the management of all other management functions: security, performance, accounting and fault” [26]. In line with configuration management is change management that ITIL describes as a systematic approach to handling all changes in a standardized method [27]. Change management not only avoids unnecessary service downtime, but it is also useful during a cyberattack. An incident response plan can be a version of change management. Similarly, strict audit logs and monitoring of logging records are IT functions which are critical to quickly recognizing attacks and obtaining details on an attack [28].

### Preventative and proactive stance

In the past, hospitals experienced difficulties with devices that refuse operating system patches or that became functionally compromised when, for example, Microsoft Windows was updated multiple times [29]. Consequently, hospitals had to delay or refrain from closing various security gaps in the operating system. There has been a recent push to promote cybersecurity as a value proposition among medical device and equipment manufacturers, shifting the approach to cybersecurity by motivating them to value it and sell it as an asset [30, 31]. Cybersecurity is not simply plugged in as an afterthought but has become one of the prerequisites of the design [32]. This has also been reinforced by the US Food and Drug Administration (FDA), that expects manufacturers to implement on-going lifecycle processes and to monitor continued safety post-market [33].

In 2017, the FDA began mandating that medical device manufacturers show that their devices are able to have updates and security patches applied throughout their lifespan. Additionally, they must show that they have addressed any undesirable issues that would affect the patients if the device was to be compromised. As part of this same regulation, the FDA requires that a “bill of materials” be shared with buyers of a medical device. The bill of materials provides transparency to the device buyer as to the source of each component (hardware and software) contained in the medical device. These new rules will apply to manufacturers, who must submit a 510(k)-pre-market submission package to the FDA [34].

These measures puts the onus on manufacturers, however, the call to approach cybersecurity with a more engaged and proactive stance should not be limited to manufacturers but should challenge health facilities as well. Hospitals ought to invest in prevention by designating resources and budgeting early, rather than depending on reactive approaches following attacks; this might be difficult in light of historic underinvestment in human resources and funding in hospital information security [35–37].

### Risk-based approach

Cybersecurity requires the highest level of security measures. However, as infallible cybersecurity is nonexistent, a risk-based approach through enterprise risk management is necessary. Even with quality IT infrastructure and practices, along with a proactive stance and information security measures, the risk of an attack will always persist. Therefore, the framework for managing cybersecurity recommended by the US National Institute of Standards and Technology (NIST) and the recommendations of the European Union Agency for Network and Information Security (ENISA) are rooted in a risk-based approach.

Risk assessment depends on the identification of at risk IT assets, stressed as the first step by the NIST Cybersecurity Framework (CSF) for critical infrastructure, and the identification of potential threats through methods such as vulnerability management [38]. An asset’s value to the organization and its exposure to risk should determine its priority in the protection processes. Quality IT is important here, as configuration management will be integral to this identification step. Risk analysis of these findings should consider tradeoffs between risks and benefits, as well as between different risks [39]. It should also evaluate the potential consequences for patient safety and maintenance of operations [38]. This requires the assessment of an incident’s impact on data and privacy protection (confidentiality), availability of information, and integrity of information. The latter is especially important as the integrity of health data can have severe consequences for the patient’s safety.

Health facilities can manage risks through various methods, from mitigating, avoiding, or transferring to accepting the risks [40]. The NIST CSF follows this identification of risks step with Protect, Detect Incidents, Respond, and Recover [40].

### Training and awareness

As humans are the weakest link in cybersecurity, health facilities’ approaches to cybersecurity should take into account the need for raising awareness among all users [41, 42]. This, of course, does not guarantee security, but it is a step in the right direction. End users, from clinicians to billing and scheduling staff, as well as patients and caregivers who connect their personal devices with the hospital network, can unintentionally—or intentionally—threaten the cybersecurity of the health facility. Human error also poses risks as in the incident at Geneva University Hospital (HUG) in October 2019 [43]. In an effort to mitigate risk, the ENISA’s Security and Resilience in eHealth publication among others recommend providing cybersecurity training [38, 44].

To offer relevant and effective trainings, health facilities should frequently assess and identify gaps in knowledge [28]. It is important for end users to realize the risks they cause through inadvertent actions. For example, they should be aware that storing data on their mobile devices can pose privacy and data-integrity risks [45], whereas the use of connected devices or removable storage devices can increase the risk of malware execution. Similarly, end users should have a concrete understanding of the threats (e.g., What is a ransomware attack, what are the effects, and how is the attack initiated?). End users are potential targets for social engineering methods, hence training programs should explore how to handle unrecognized e-mails and avoid phishing tactics, while encouraging basic digital-hygiene practices (e.g., strong passwords, not clicking on unknown links).

Cyberattacks, such as the May 2017 worldwide WannaCry attack, serve as a wakeup call, but it is in the best interest of organizations to keep up vigilance even when threats are not in the headlines [46]. One way to do this is by enacting mock exercises and simulating cybersecurity drills. Health facilities can approach this in different ways: from having the information security team send users simulated phishing e-mails, to setting up drills for IT officers such as locating and neutralizing unauthorized devices on the network [47, 48]. These exercises can even evaluate the effectiveness of the organization’s current training programs [49].

## Recommended Cybersecurity measures

### Vulnerability management, patch management

Exposure and vulnerability management involves the identification, evaluation, and mitigation of IT vulnerabilities. It relies heavily on threat-monitoring processes but also entails all the identification steps: risk assessment, remediation or mitigation steps, and reevaluation [50]. In handling and investigating attacks and post-infection remediation, Endpoint Detection and Response (EDR) solutions should be used. In most cases, this risk assessment is highly complex. Among the steps towards remediation or mitigation, there is also patch management that can become complicated by a health facility’s need to operate 24/7/365. Risk analysis is at the core of patch processes: weighing the sensitivity of data on the server and an enterprise’s critical functions or assets vulnerable to an attack [26].

Organizations should actively search out vulnerabilities in their systems and maintain ongoing vulnerability management with penetration testing [28]. Early detection can help reduce exposure to a security risk. The identification of vulnerabilities should also be followed with configuration hardening or patch processes without an overemphasis on zero-day vulnerabilities. Gartner analysts recently found that 99% of exploits are based on vulnerabilities that were known to security and IT professionals for over six months [51]. In prioritizing the remediation of different vulnerabilities, organizations should consider such findings.

As for the importance of maintaining quality IT infrastructure, configuration management has the benefit of increasing ease in assessing vulnerabilities because of a broader understanding of the facilities’ IT infrastructure and in running risk assessments, as well as analyses required for patch processes. Patching should be applied to all systems in the configuration (this includes the operating system and third-party applications) and changes should be noted by change management [50].

### Administrative privileges and administrative multifactorial authentication

The risks associated with granting administrative privileges to users in health facilities are immense. According to CyberSheath’s APT Privileged Account Exploitation report, the vast majority of large-scale attacks that caused significant damage and expenses were initiated through the compromise of a privileged account such as that of a third-party provider [52]. This was the case for the attack that took place at Hancock Regional Hospital in January 2018, when the login credentials to a vendor’s account were compromised [23].

Health entities should grant administrative privileges in a controlled and restrictive manner, in order to minimize the number of such accounts to an enterprise-dependent manageable sum [28, 53]. These accounts should be inventoried, monitored for abnormal use, and evaluated for log entries. To avoid malicious insider threats, the health entity should also enforce local password policy and revisit their criteria for privileged access in addition to the vetting of users. A study revealed that disgruntled employees account for 70% of computer-related criminal activity [54]. Organizations should address the risk of such threats by closely monitoring the lifecycle of user accounts and revoking client and user certificates when no longer in use. Additionally, end users requiring administrative privileges should have two accounts: one that has privileges limited to local machines and another with no administrative privileges to be used for routine tasks such as browsing the internet or checking emails [28, 47, 55]. When necessary, direct web-access on critical devices should be denied or the use of encapsulated browsers should be enforced.

It is important to provide users who are granted administrative or privileged accounts with additional training on the risks brought on by their privileges, as it is important to equip them with the proper security measures. Among the most important measures is the use of multifactorial authentication for all administrative and privileged users—preferably for all users. The Center for Internet Security’s (CIS’s) Critical Security Controls for Effective Cyber Defense lists the use of smart cards, One Time Passwords, or biometrics, among the techniques to implement this vital step [28].

### Incident response plan

As cyberattacks have become increasingly frequent and consequential in recent years, health facilities should prepare an incident response and business continuity plan. These plans should be regularly tested, exercised, and stored offline [55]. Plans should involve an agreed upon process with the appropriate stakeholders identified. It is important to have a designated team and a cybersecurity leader, or simply a designated person in cases where the organization does not have a CISO [56, 57]. The roles and responsibilities should be clearly divided within the team. The organizations should also have an agreement on what constitutes as a reportable incident and when to escalate [58, 59]. Ideally, plans should embed prevention training as well.

Incident response plans should also endorse post-incident steps. This can involve enforcing organization-wide password resets after an attack, factory resetting, and replacing compromised hardware and software as necessary. However, there needs to be an internal plan for regrouping and implementing changes [40]. The IT and cybersecurity system and its management should then be adapted to the new needs and requirements that were revealed by the incident (i.e., patching and beyond).

A notification system should be established between the health facility and the manufacturers [60]. A process can be built for those in the enterprise (e.g., clinicians, business administrators, and IT staff) to report incidents directly to the manufacturers. In fact, this type of sharing is also being mandated in the most recent FDA 510(k) pre-market submission guidelines [34].

### Information sharing

The exchange of potential threats, indicators of compromise, best practices, vulnerabilities, lessons learned, and of mitigation strategies between stakeholders across public and private sectors is an essential step in building the cybersecurity of healthcare systems [61, 62]. Information sharing facilitates situational awareness and a solid understanding of threats and threat actors, their motivations, campaigns, tactics, and techniques. Consequently, it better equips decision makers to understand organizational exposure and to employ enterprise risk management policies. Information sharing should include all stakeholders: providers, manufacturers, suppliers, payers, and electronic record providers, as well as government(s) where applicable.

There are organizations that exist specifically to facilitate collaboration between institutions, for example, the National Health Information Sharing and Analysis Center (NH-ISAC), a global, member-driven non-profit providing a forum for trusted sharing amongst healthcare organizations. The EU adopted the Network and Information System (NIS) Directive in 2016—the first EU law specifically focused on cybersecurity—to be transposed by member states by 2018. The directive requires member states, most notably, to adopt national cybersecurity strategies, to designate national competent authorities, and to develop one or more computer security incident response teams (CSIRTs). It also establishes security and incident notification requirements for “operators of essential services,” such as healthcare organizations, even requiring incidents of certain magnitudes to be reported to national authorities. To promote swift and effective operational cooperation regarding threats and incidents, the directive emphasizes coordination among member states, setting up a CSIRT network (also to include CERT-EU), and a strategic NIS “cooperation group” to support and facilitate cooperation and information exchange among member states [63].

### Privacy-conscious data sharing and processing

The sharing of medical and genomic data, across departments and institutions, is necessary for both effective patient care and for meaningful research that advances the state-of-the-art in personalized medicine. In fact, the recent increasing trend towards P4 (Predictive, Preventive, Personalized and Participatory) medicine is called to revolutionize healthcare by providing better diagnoses and targeted preventive and therapeutic measures. However, clinical and research data on large numbers of individuals must be efficiently shared among all stakeholders. In this context, cybersecurity is as relevant as it is in regular hospital operations, but the privacy risks that stem from disclosing medical and genomic data play a prominent role and have become a barrier in the advancements of P4 medicine [64]. This is further reflected in the evolution of stricter regulations (e.g. HIPAA in US and GDPR in the EU [9, 11]).

The challenges of privacy-conscious data sharing and processing can be addressed through the use of advanced cryptographic mechanisms (such as homomorphic encryption [65, 66], trusted hardware [67], secure multiparty computation [68, 69]), and strong trust distribution techniques (such as distributed ledger technologies [70]). The use of these technologies provides security guarantees beyond those implemented by traditional approaches against cyberattacks [71], with the following four direct advantages: (a) achieving a more fine-grained control on access permissions, hence reducing or avoiding the need of privileged accounts to third parties, (b) implementing minimization principles on the released data for the agreed usage, in line with the latest and stricter data protection regulations and minimizing the risk of breaches and intentional or unintentional data misuse, (c) keeping individual and identifiable data within the confines of the security perimeter of the medical institution that governs them, and (d) enabling distributed logging and access control management, hence avoiding single points of failure and greatly reducing the effect of a breach and the risk of a successful attack, while allowing for more advanced implementations of auditability, accountability and incident recovery. Consequently, privacy-conscious data sharing and processing approaches are aligned with the aforementioned risk-based cybersecurity strategies, provide guarantees that go beyond the latter, yet enables operations across medical institutions that would otherwise be impossible.

## Recommendations for connected medical devices

The FDA defines medical devices as

An instrument, apparatus, implement, machine, contrivance, implant, in vitro reagent, or other similar or related article, including a component part, or accessory [ … ] intended for use in the diagnosis [ … ] cure, mitigation, treatment, or prevention of disease [ … ] [72].

This definition encompasses equipment such as beds, in-house treadmills, intravenous pumps, and monitors, as well as implantable and connected devices such as pacemakers and insulin pumps. Additionally, wearable devices (such as Fitbits) that monitor, and record health and lifestyle data can now be connected to clinicians’ devices. These devices can propagate flaws or incidents in cybersecurity and act as weak elements in the security chain by which malware can spread. The diversity in devices can also make it difficult to enact strict security policy, but the cybersecurity of these devices is critical. Medical devices are typically in direct contact with patients and can increase risks to hospital operations and patient safety.

Advancements such as the Internet of Things enables remote medical care and precision in healthcare delivery. However, clinical care utility and safety need to be balanced with security and privacy. Devices are highly interconnected in the hospital network and large sums of collect clinical data that need to be securely transferred, but these devices also have inherent limitations that expose them to vulnerabilities. They often do not have the proper security measures because they do not have the battery power or the built-in resources to efficiently employ security measures such as encryption and forensic processes, threat modeling activities, and malware detection [58, 60]. Devices designed to function in isolation often end up integrated into the network, whereas physical security of the wearable devices is nearly impossible as they do not typically have long life spans and their operating system or relevant platforms become outdated relatively quickly [56, 58].

Decision makers should evaluate the expected lifetime of devices (e.g., manufacturer/vendor-support or operating system-support) before purchase. In conjunction, equipment maintenance is critical to medical-device security. Hospitals and manufacturers, with support from certifying authorities, should develop a patching policy that minimizes equipment downtime and enables timely updates through a collaboration with the external manufacturing community and internal stakeholders. Collaboration with manufacturers can allow facilities to better monitor new alerts in order to keep up with critical or urgent patches and updates. Facilities should also develop and budget for life-cycle management in order to retire devices that cannot be replaced right away.

It is also essential for IT to maintain a regularly updated inventory of all devices on the network (authorized and unauthorized). Hospital networks often have numerous personal devices that are integrated. Patients and physicians often connect external mobiles and wearables [73], thus increasing exposure and complicating bring your own device (BYOD) policies. The health organization should enact reasonable measures and policies to block connectivity of unapproved personal devices (mobiles, tablets …) [55], even using mobile device management or software distribution systems. Besides this, health facilities should enforce local data encryption, when possible, in a preventative stance.

## Conclusion

A year and a half after this workshop, attacks on hospitals continue to take headlines. At the beginning of October 2019, three hospitals in Alabama (US) faced a ransomware attack that forced them to diverge new patients to nearby hospitals [74]. Around the same time, another ransomware infection on seven Australian hospitals was reported [74]. There continues to be an outbreak of these attacks, further stressing the urgency of the matter at hand.

Building the cyber resilience of a hospital is vital and it is a shared responsibility. Users (i.e., clinicians and administration staff) should undergo training and should practice digital hygiene, decision makers should enforce the proper policies and consider cybersecurity in purchasing decisions, and manufacturers should equip their products with the appropriate cybersecurity measures. The information security teams of hospitals should also enact and upkeep the proper tools to safeguard the hospital and patients.

Information security teams should equip users to counter social engineering methods by, for example, filtering e-mail content, auto-checking suspicious URLs in e-mails for linked malicious code, whitelisting trustworthy websites and applications, as well as blocking Flash, advertisements and untrusted JAVA code on the Internet, as necessary [55]. Other tactics for reducing exposure should be used, such as intentionally changing default passwords and regularly updating security configurations on laptops, servers, workstations, firewalls, etc. [47]. Antivirus software is also important, along with penetration tests, control of physical access, and the maintenance of regularly updated backups (which should be stored offline). The organization’s website and the industrial control systems, including HVAC, cameras, fire alarm panels, should be secure and locked down from attacks. EDR Software can also help detect malware breaches and react properly to recorded infections. Finally, there should be appropriate tools in place for protecting data shared across different departments or medical institutions in a privacy-conscious way, therefore reducing the risk of intentional or unintentional breaches through trust distribution [64].

Cybersecurity is also a matter of arbitrating tradeoffs [39]. As mentioned, utility and safety need to be balanced with security, privacy, and compliance with data protection regulations, especially in the highly distributed and collaborative environments required for precision medicine. Yet, convenience cannot be left out of the equation. Without considering the latter point, these recommendations will remain theoretical and inapplicable in actual practice. A physician who wants to store or access clinical data on their mobile phone is not doing so to increase exposure to cyber threats but for the sake of convenience and efficiency in the delivery of care, and the quality of care. Similarly, an information security officer who takes a system offline to apply updates or patches does not intend to inconvenience health providers but to decrease the risks against unexpected downtime from large-scale attacks. There should not be two sides working independently of each other towards their own goals, but a collective, multidisciplinary team working towards protecting and improving patient care and data.

## Additional resources

Cybersecurity of healthcare organizations is critical to patient safety, as well as to hospital operations. Many resources have become available in recent years. Here are some:

- ISO/IEC 27002 (2013)
- CIS Critical Security Controls for Effective Cyber Defense (2016)
- ENISA Security and Resilience in eHealth: Security Challenges and Risks (2015)
- Medical Device Innovation Safety and Security Consortium (MDISS.org)
- DTS Cybersecurity Standard for Connected Diabetes Devices (www.dtsec.org

## Data Availability

Data sharing is not applicable to this article as no datasets were generated or analyzed during the current study.

## Abbreviations

PII: Personally identifiable information
PHI: Protected health information
HIPAA: Health Insurance Portability & Accountability Act
US: United States
EU: European Union
GDPR: General Data Protection Regulation
EHR: Electronic health records
HVAC: Heating, ventilation, and air conditioning
CERT: Computer Emergency Response Team
CISO: Chief Information Security Officer
CIO: Chief Information Officers
ITIL: Information Technology Infrastructure Library
IT: Information technology
FDA: Food and Drug Admiration
NIS: Network & Information Systems
NIST: National Institute of Standards and Technology
ENISA: European Union Agency for Network and Information Security
CSF: Cybersecurity Framework
HUG: Geneva University Hospital
EDR: Endpoint Detection and Response
NH-ISAC: National Health Information Sharing and Analysis Center
P4: Predictive, Preventive, Personalized and Participatory
BYOB: Bring your own device
